# Metformin limits the adipocyte tumor-promoting effect on ovarian cancer

**DOI:** 10.18632/oncotarget.2012

**Published:** 2014-05-26

**Authors:** Calvin Tebbe, Jasdeep Chhina, Sajad A. Dar, Kalli Sarigiannis, Shailendra Giri, Adnan R. Munkarah, Ramandeep Rattan

**Affiliations:** ^1^ Division of Gynecology Oncology, Department of Women's Health, Obstetrics and Gynecology, Henry Ford Hospital, Detroit, MI 48202, USA; ^2^ Department of Neurology, Henry Ford Hospital, Detroit, MI, USA

**Keywords:** ovarian cancer, 3T3L1 adipocytes, metformin, AMPK, ID8 cells, adipogenesis

## Abstract

Omental adipocytes promote ovarian cancer by secretion of adipokines, cytokines and growth factors, and acting as fuel depots. We investigated if metformin modulates the ovarian cancer promoting effects of adipocytes. Effect of conditioned media obtained from differentiated mouse 3T3L1 preadipoctes on the proliferation and migration of a mouse ovarian surface epithelium cancer cell line (ID8) was estimated. Conditioned media from differentiated adipocytes increased the proliferation and migration of ID8 cells, which was attenuated by metformin. Metformin inhibited adipogenesis by inhibition of key adipogenesis regulating transcription factors (CEBPα, CEBPß, and SREBP1), and induced AMPK. A targeted Cancer Pathway Finder RT-PCR (real-time polymerase chain reaction) based gene array revealed 20 up-regulated and 2 down-regulated genes in ID8 cells exposed to adipocyte conditioned media, which were altered by metformin. Adipocyte conditioned media also induced bio-energetic changes in the ID8 cells by pushing them into a highly metabolically active state; these effects were reversed by metformin. Collectively, metformin treatment inhibited the adipocyte mediated ovarian cancer cell proliferation, migration, expression of cancer associated genes and bio-energetic changes. Suggesting, that metformin could be a therapeutic option for ovarian cancer at an early stage, as it not only targets ovarian cancer, but also modulates the environmental milieu.

## INTRODUCTION

Ovarian cancer is the fifth leading cause of cancer death in women and the most fatal of all gynecological cancers [1]. This high mortality has been attributed to the presence of widespread peritoneal metastasis at the time of diagnosis [2]. A unique feature of ovarian cancer is its propensity to peritoneal spread, where it seems to favor the omentum as one of its first metastatic sites [3-5]. At the time of diagnosis, over 60% of patients with epithelial ovarian cancer present with an omentum loaded with tumor [3, 6]. The omentum is largely composed of adipocytes, and it is these adipocytes that have been recently implicated as major players in attracting the ovarian tumor cells [6]. It was recently demonstrated that omental adipocytes bait the ovarian tumor cells by a slew of adipokines and help them in metastasizing by bestowing a proliferative advantage and supply of fatty acids as fuel [6]. Similar to omental adipocytes, the adipose tissue at other sites has been shown to create a favorable microenvironment for breast [7], gastric [8], and colon cancers [9]. Adipocytes have been established as active ‘endocrine organs’ that secrete adipokines and cytokines in response to external stimuli and provide metabolic and immune regulatory functions [10]. These features enable the adipocytes to not only support the tumor growth but also engage in a cross-talk with the tumor cells.

Metformin, the most prescribed drug for Type II diabetes, has been found to possess anticancer properties. This has been shown in retrospective studies on breast [11], prostate [12], colorectal [13], pancreatic [14], endometrial [15] and ovarian malignancies [16]. Numerous preclinical investigations have also demonstrated that metformin inhibits growth of various cancer cells, including those arising from breast [17], endometrium [18], ovary [19], brain [20], lung [21], prostate [22], pancreas [23], kidney [24], and colon [25]. We have recently shown the efficacy of metformin alone and in combination with cisplatin in reducing ovarian cancer growth both in vitro and in vivo [19, 26]. We have also shown association of metformin intake with better survival in a cohort of ovarian cancer patient population [27]. Additionally, metformin has been shown to ameliorate obesity and ageing by targeting common pathways associate with cancer, which could potentially be translated to its cancer preventive potential [28-31]. Because of the widespread laboratory and clinical based evidence, approximately 182 clinical trials investigating metformin's potential in human patient populations are currently ongoing [http://www.clinicaltrials.gov/].

The mechanism of action of metformin has been shown to be mainly due to the activation of a master metabolism regulator, adenosine monophosphate activated kinase (AMPK), via its upstream kinase, liver kinase B1 [32]. AMPK is a hetero-trimeric kinase that along with regulation of metabolic pathways also participates in various signaling pathways and controls protein expression [33]. Its anticancer effects are mainly carried out by inhibition of the mammalian target of rapamycin (mTOR) pathway and cell cycle [19, 25, 34]. It has also been shown to inhibit tumors through indirect effects of lowering glucose, insulin and insulin growth factor-1 levels [35, 36]. While recent reports have shown that metformin can also act in AMPK-independent manner [22, 37], most of its manifold effects can still be attributed to activation of AMPK. Most of the studies on cancer cells have focused more on the signaling effects of metformin and AMPK activation, while not much attention has been given to the vast metabolic changes that are associated with AMPK activation. AMPK by virtue of its metabolic regulatory effect also regulates adipogenesis and lipolysis [38, 39], placing it in a unique position to alter the adipocyte generated microenvironment for the tumor cells. Both adipogenesis and lipolysis have been shown to aid the ovarian tumor cells in proliferation and metastasis [6]. Recently, an effect of obesity (adiposity) on cancer promotion has been in focus. Conditions of increased adiposity results in an increased in mTOR signaling, inflammatory factors and growth factors like insulin and IGF-1. Metformin has been reported to inhibit almost all of these propagators in models of obesity, aging and cancer making it as a good agent to target adipocyte specific tumor enhancing effects [28].

Recent research suggests that ovarian cancer cells use omental adipocytes as their first repository to derive energy and multiply in number in preparation for further growth and spread [6]. Therefore, it would be rational to design and test therapies that can target this first step in the ovarian cancer metastasis and may prevent its further spread. With this objective, we investigated whether metformin can regulate the adipocyte-adipokine stimulated ovarian cancer growth and therefore impact the initial steps of ovarian cancer metastasis. Overall, our study shows that metformin has the ability to inhibit adipogenesis and the ovarian tumor promoting effects driven by adipocytes.

## RESULTS

### Metformin inhibited adipocyte induced proliferation of ID8 ovarian cancer cells

**Figure 1:**
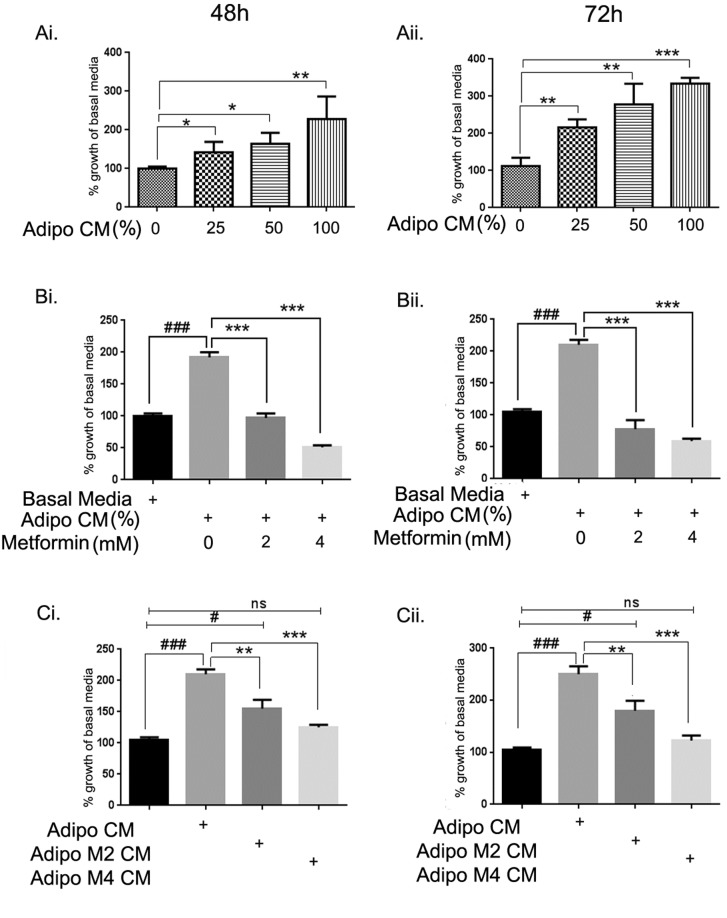
Metformin inhibited adipocyte driven growth of ID8 ovarian cancer cells 3T3L1 mouse preadipocytes were differentiated into adipocytes over a period of 9 days and conditioned media (CM) was collected and used to treat ID8 cells. Mouse ovarian cancer cell line ID8 was exposed to the adipocyte CM (adipo CM) at various dilutions (0-100%) in basal media and MTT assay performed at 48 (Ai) and 72 (Aii) hours (*p < 0.05, **p < 0.01, ***p < 0.001 compared to 0, basal media). ID8 cells were exposed to 50% adipo CM in presence of 2 and 4 mM concentration of metformin. Cell proliferation was assessed by MTT assay performed at 48 (Bi) and 72 hours (Bii) (###p<0.001 compared to basal media; ***p < 0.001 metformin treated compared to adipo CM alone). ID8 cells were exposed to the 50% adipo CM or 50% CM from adipocytes that were treated with different concentrations of metformin while differentiating (adipo M2 CM: metformin 2 mM and adipo M4 CM: metformin 4 mM). Cell proliferation was assessed by MTT assay performed at 48 (Ci), and 72 (Cii) hours (###p < 0.001, #p < 0.05, ns: non-significant compared to basal media; **p < 0.01, ***p < 0.001 metformin treated compared to adipo CM alone).

To examine the effect of adipocyte conditioned media (adipo CM) on the growth of ID8 mouse ovarian tumor cells, ID8 cells were exposed with Rosewell Park Memorial Institute (RPMI) media and adipo CM at various ratios (75:25; 50:50; 0:100). A dose dependent increase in proliferation was observed at 48 and 72h compared to the cells cultured in basal media (mix of RPMI and Dulbecco's modified Eagle's medium [DMEM]) (Fig. 1Ai & Aii). Treatment of ID8 cells with metformin at low concentrations (2-4 mM) inhibited the adipo CM mediated increased proliferation of ID8 cells at both time points (Fig. 1Bi & Bii). CM from adipocytes differentiated in presence of metformin (2 and 4 mM) significantly decreased proliferation of ID8 cells (Fig. 1Ci & Cii). These data indicate that adipo CM can accelerate the growth of ovarian cancer cells and this effect can be attenuated by metformin treatments, either to the differentiating adipocytes or to the ID8 cells.

### Metformin inhibited adipocyte induced migration-invasion of ID8 ovarian cancer cells

**FIGURE 2:**
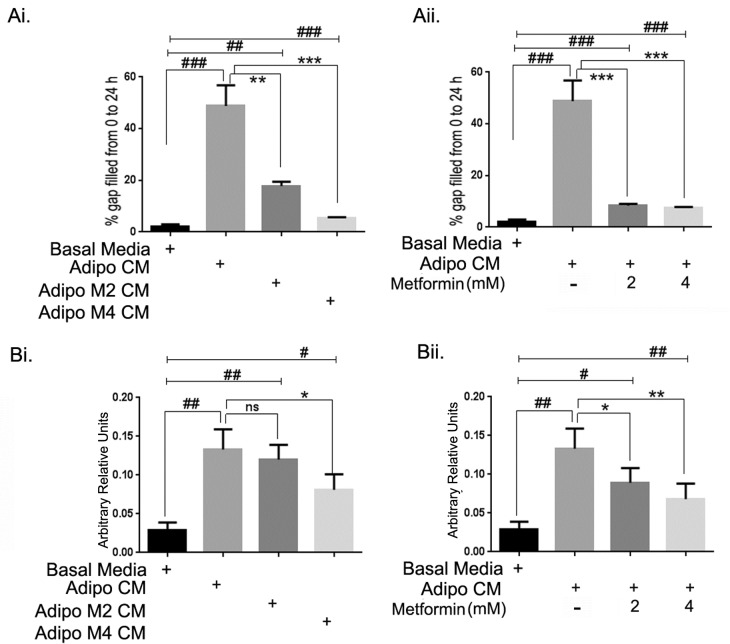
Metformin inhibited adipocyte induced migration-invasion of ID8 ovarian cancer cells (Ai) ID8 cells grown to confluence were kept in low serum conditions (0.2%) overnight and scratch assay was performed as described in the Methods section. The media was replaced by respective conditioned media as indicated with adipocyte CM (adipo CM) and CM from adipocytes differentiated in presence of metformin 2 or 4 mM (adipo M2 CM or adipo M4 CM). The pixel distance between the gap was measured at a designated point at 0 and 24 hours. The distance covered at 24 hours was subtracted from the 0 time point to estimate the area covered by the migrating cells (###p < 0.001, ##p < 0.01 compared to basal media; **p < 0.001, ***p < 0.0001 compared to adipo CM). (Aii) ID8 cells were treated with metformin (2 or 4mM) in presence of adipo CM. Migration was measured for all groups (###p < 0.001, ##p < 0.01 compared to basal media; **p < 0.001, ***p < 0.0001 compared to adipo CM). (Bi) Invasion of ID8 cells towards adipo CM and adipo Met CM (2 and 4mM) was estimated using Boyden Chamber (##p < 0.01, #p < 0.05 compared to basal media; **p < 0.001, *p < 0.05, ns: non-significant compared to adipo CM alone). (Bii) ID8 cells were treated with metformin (2 or 4mM) in presence of adipo CM and invasion of ID8 cells was estimated (##p < 0.01, #p < 0.05 compared to basal media; **p < 0.001, *p < 0.05, ns: non-significant compared to adipo CM).

We next examined the effect of adipo CM on cell migration and invasion using scratch and Boyden chamber migration and invasion assays, respectively. As shown in Figure 2Ai, adipo CM significantly increased (~ 50 fold) the migration of ID8 cells. However, metformin treated adipocyte CM (adipo Met CM; metformin 2 and 4 mM) had significantly less effect on cell migration (Fig. 2Ai). Treatment of the ID8 cells with metformin also inhibited adipo CM mediated ID8 cell migration (Fig. 2Aii). For assessing invasion, Boyden chamber migration and invasion assay was used as described in the methods. Invasion of ID8 cells was increased ~ 4-fold in response to adipo CM (Fig. 2Bi), while the invasion was less in response to adipo Met CM at the higher dose of metformin (4 mM) (Fig. 2Bi). Metformin treatment of ID8 cells at both doses inhibited the adipo CM mediated invasion of ID8 cells (Fig. 2Bii). These data suggest that adipocytes have a profound effect on the migration and invasion of ID8 cells and metformin treatment can attenuate both migration and invasion of ovarian cancer cells facilitated by adipocytes.

### Low dose of metformin did not have a direct effect on ID8 cells

Metformin has been shown by us and others to inhibit proliferation of ovarian cancer cells [19, 40]. To confirm that the inhibitory effects of metformin at 2 and 4 mM on ID8 proliferation, migration and invasion are specific to counteract the adipo CM mediated effect and not a direct effect on the ID8 cells, ID8 cells were treated with various doses of metformin (1-5 mM) and cell proliferation was assessed by metformin, trypan blue, 3-(4,5-dimethylthiazol-2-yl)-2,5-diphenyl tetrazolium bromide (MTT) assay. Metformin did not result in any significant changes in proliferation at 48 or 72 hours at any dose (Fig. S1Ai & Aii). Furthermore, metformin did not affect migration (Fig. S1B) or invasion (Fig. S1C) of ID8 cells, induced by 10% FBS as seen by scratch assay and Boyden Chamber assay, respectively. Thus, the inhibitory action of metformin seen in ID8 in presence of adipo CM is specific to that driven by adipocytes.

### Metformin treatment inhibited adipogenesis

**FIGURE 3:**
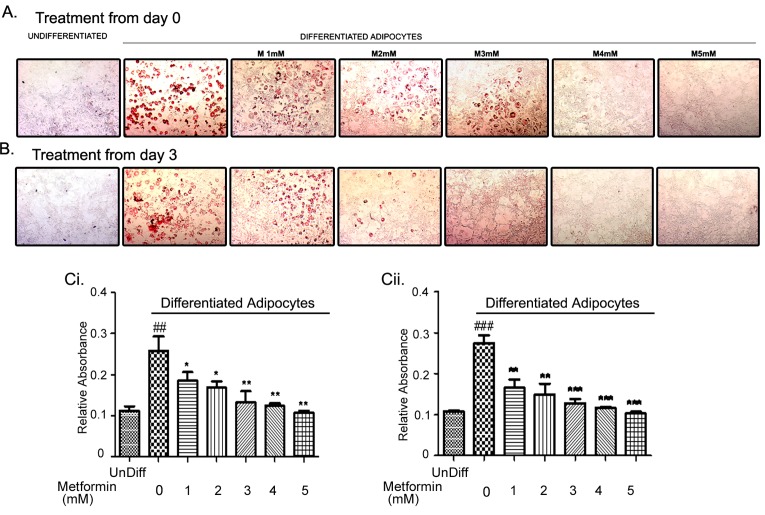
Metformin inhibited adipocyte differentiation 3T3L1 mouse preadipocytes were differentiated over a period of 9 days. One set was treated from day 0 (A) or day 3 (B) with various concentrations of metformin (1-5mM). Differentiation of adipocytes was monitored by accumulation of lipid droplets visualized by Oil Red O staining. The accumulated lipid droplets were quantified by coloriemeteric absorbance (Ci, Cii) (##p < 0.001 compared to undifferentiated preadipocytes (undiff); *p < 0.05, **p < 0.01, ***p < 0.001 compared to Diff: untreated differentiated adipocytes).

Since metformin can inhibit lipid biosynthesis by activation of AMPK [19, 41], we investigated whether metformin would alter the differentiation of adipocytes. For this goal, mouse preadipocytes cell line (3T3L1) was differentiated as described in the methods. Metformin was added at various time points during 3T3L1 differentiation process including day 0, 3, or 5. Adipocyte differentiation was examined on day 9 by Oil Red O staining as evident by red stained numerous lipid droplets. Addition of metformin from day 0 (Fig. 3A) or day 3 (Fig. 3B) of differentiation resulted in a dose dependent inhibition of lipid accumulation. Quantitative analysis of the lipid content reflected a significant inhibition of lipid droplet accumulation in dose dependent manner (Fig. 3Ci & Cii). Addition of metformin on day 5 of the differentiation had no effect on the number of lipid droplet accumulation (data not shown). Furthermore, metformin at low concentrations (1-4 mM) also inhibited the proliferation of preadipocytes (Fig. S2), confirming that metformin can attenuate the expansion of preadipocytes and also effectively inhibit the conversion of preadipocytes to adipocytes.

### Metformin inhibited adipogenesis by regulating transcription factors and activating AMPK

**FIGURE 4:**
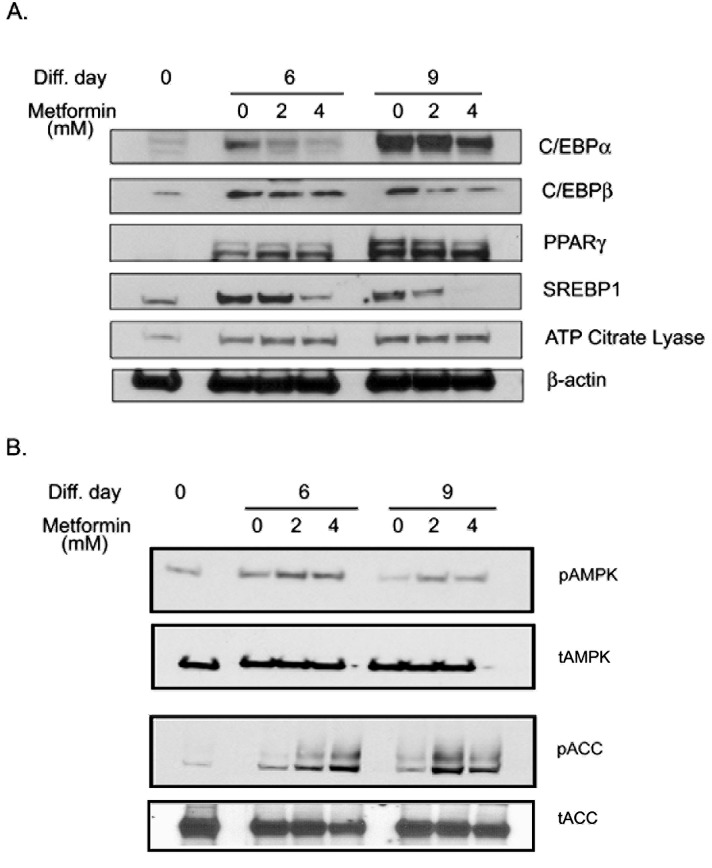
Metformin inhibited adipogenesis regulating transcription factors and activates AMPK Protein was isolated from differentiating adipocytes on days 0, 6 and 9 in the presence or absence of metformin (2 and 4 mM). An equal amount of protein (50μg) was loaded and separated using SDS-PAGE. (A) Protein expression of CCAAT-enhancer binding protein alpha (CEBPα), CCAAT-enhancer binding protein beta (CEBPß), Peroxisome proliferator-activated receptor gamma (PPARγ), Sterol regulatory element-binding protein −1 (SREBP1) and adenosine triphosphate (ATP) Citrate Lyase were detected by immunoblot analysis using their specific antibodies. Beta-actin was used as control for equal protein loading. (B) Same protein samples were also immuno-blotted for expression of phosphorylated (p) and total (t) adenosine monophosphate activated kinase (AMPK) and acetyl CoA carboxylase (ACC). All blots are representative of 2 individually performed experiments.

Adipogenesis is regulated by coordinated activation of various transcription factors [42]. To understand the molecular mechanism of metformin mediated inhibition of adipogenesis, the effect of metformin on the expression of key transcriptions factors was examined at 2 time points (day 6 and 9) during adipocyte differentiation. CCAAT-enhancer binding protein alpha (CEBPα) was increased from day 0 to 6 to 9 of differentiation and inhibited by metformin treatment. CCAAT-enhancer binding protein beta (CEBPß), expression also showed a similar pattern (Figs. 4A & S3B). Maximal sterol regulatory element-binding protein −1 (SREBP1) expression was reached on day 6, and plateaued through day 9. It was inhibited by metformin at both time points in a dose dependent response (Figs. 4A & S3D). Peroxisome proliferator-activated receptor gamma (PPARγ) expression was also increased from day 0 to 6 to 9 of differentiation, but metformin had no effect on its expression (Figs. 4A & S3C). Similarly, metformin did not modulate the expression of adenosine triphosphate (ATP) citrate lyase (Figs 4A & S3E). Densitometric analysis of the blots reflects the time dependent changes in expression profiles (Fig. S3). Metformin treatment induced AMPK activation at both day 6 and day 9 as seen by increased phosphorylated form of AMPK and its immediate downstream target phosphorylated acetyl CoA carboxylase (ACC; Figs. 4B & S3F, G). These data indicate that metformin treatment activates and maintains the activated state of AMPK, which correlates with inhibition of key adipogenesis transcription factors and subsequent halting of preadipocyte differentiation to adipocytes.

### Metformin inhibited adipocyte mediated lipid accumulation in ID8 cells

**FIGURE 5:**
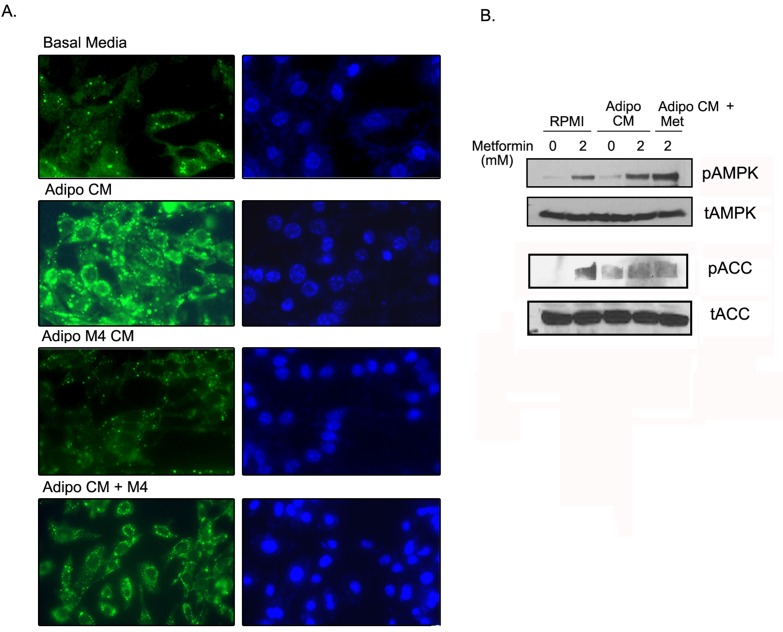
Metformin inhibited adipocyte mediated lipid accumulation in ID8 cells (A) ID8 cells were grown in 50% adipocyte CM (adipo CM) or CM from adipocytes differentiated in presence of metformin 2 or 4 mM (adipo M4 CM, or adipo CM) in the presence of metformin (adipo CM + M4) for 72-96 hours in chamber slides. The cells were stained with Bodipy 493/503 and counterstained with Hoechst 33342 as described in the Methods. The lipid droplets were stained green and visualized with a fluroscent microscope at 40x magnification. (B) Under same conditions, the protein extracted was subjected to western blot and immuno-blotted for expression of phosphorylated (p) and total (t) adenosine monophosphate activated kinase (AMPK) and acetyl CoA carboxylase (ACC). Micrographs and blots are representative of 2 individually performed experiments.

To investigate if metformin can limit the lipid droplet formation in ID8 cells by adipocytes, we exposed the ID8 cells to adipo CM and stained for lipid droplets using the Bopidy stain. Adipo CM induced robust formation of lipid droplets in the ID8 cells (Fig. 5A, second panel), while adipo Met CM induced minimal lipid accumulation (Fig. 5A, third panel). Treating the ID8 cells with metformin (4 mM), reduced adipo CM mediated lipid accumulation but did not inhibit it completely (Fig. 5A, last panel). Metformin treatment induced the phosphorylation of AMPK and ACC in ID8 cells (Fig. 5B, lanes 1 & 2) in presence of adipo CM (Fig. 5B, lanes 3 & 4). ID8 cells exposed to adipo Met CM (metformin 2mM) also showed increased phosphorylation of AMPK and ACC (Fig. 5B, lane 5). These data indicate that metformin treatment to the differentiating adipocytes or the ID8 cells results in the activation of AMPK pathway which correlates with diminished lipid accumulation in the cancer cells.

### Metformin attenuated expression of adipocyte induced tumor promoting genes

**FIGURE 6:**
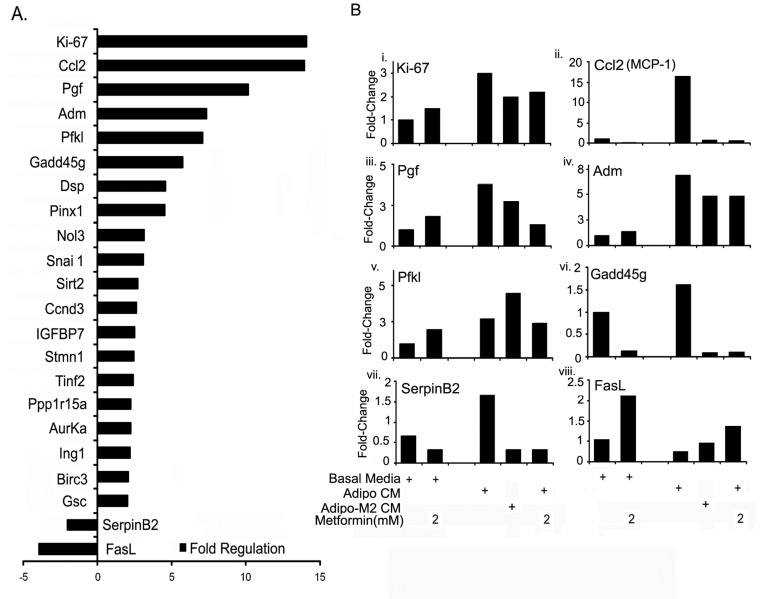
Metformin inhibited expression of adipocyte induced tumor promoting genes (A) ID8 cells were exposed to adipo CM for 24 hours before extracting RNA and synthesizing complementary DNA (cDNA). The cDNA was applied to pre-made Mouse Cancer PathwayFinder™ RT² Profiler™ PCR Array (SA Biosciences). (A) Twenty-two genes modulated by 2-fold or more in ID8 cells exposed to adipocyte conditioned media (adipo CM). Ki-67, chemokine (c-c motif) ligand 2 (Ccl2, also known as MCP-1, monocyte chemotactic protein); Placental growth factor (Pgf); Adrenomedullin (Adm); Phosphofructo kinase, liver (Pfkl); Growth arrest and DNA-damage-inducible 45 gamma (Gadd45g); Desmoplakin (Dsp); PIN2/TERF1 interacting, telomerase inhibitor 1 (Pinx1); Nucleolar protein 3 (apoptosis repressor with CARD domain [Nol^3^]); Snai1 (Snail homolog 1 [Drosophila]); Sirtuin 2 -silent mating type information regulation 2 (Sirt2); CyclinD3 (CcnD3); Insulin growth factor binding protein 7 (IGFBP7); Stmn1; Tinf2 (Terf1 [TRF^1^]-interacting nuclear factor 2); Protein phosphatase 1, regulatory (inhibitor) subunit 15A (Ppp1r15a); aurora kinase A (AurKa); Inhibitor of growth protein 1 (Ing1); baculoviral IAP repeat-containing 3 (Birc3); goosecoid homeobox (Gsc); Serine [or cysteine] peptidase inhibitor, clade B, member 2 (SerpinB2); Fas ligand (FasL), tumor necrosis factor superfamily, member 6. (B) ID8 cells in basal media with or without metformin and in adipo CM with or without metformin were used to extract RNA and synthesize cDNA after 24 hours to validate the top 6 modulated genes. Ki-67 (Bi), Ccl2 (MCP-1) (Bii), Pgf (Biii), Adm (Biv), PFKl (Bv), Gadd45g (Bvi), SerpinB2 (Bvii) and FasL (Bviii).

To further examine the mechanism of adipocyte mediated cancer cell growth and metformin effect, we used the Mouse Cancer PathwayFinder™ RT² Profiler™ PCR Array to ascertain the changes in tumor promoting genes occurring in ID8 cells exposed to adipocyte CM that could be responsible for its pro-tumorigenic effects. Out of the 84 genes explored (Fig. S4), 22 genes were found to be differentially modulated by more than 2-fold (Fig. 6A). Out of these 22 genes, 20 were up-regulated and 2 were down-regulated (Fig. 6A). The modulated genes observed were spread across the 9 cancer pathways, which included angiogenesis, apoptosis, cell cycle, cell senescence, DNA damage and repair, epithelial to mesenchymal transition (EMT), hypoxia, metabolism and telomeres and telomerase (Fig. S4). We next tested the effect of metformin treatment on ID8 cells on the expression of these 22 genes in the presence of adipo CM and adipo Met CM. The top 6 highly up-regulated and the 2 down-regulated genes are depicted in Figure 6B, while the rest are presented in a supplementary figure (Fig. S5). Mouse Ki-67 (mKi-67), the most highly up-regulated gene in the array (~ 14 fold), was found to be up-regulated 3-fold on validation and showed an inhibitory trend under metformin treatment both when the treatment was made to the differentiating adipocytes (second last bar) or when the ID8 cells were exposed to adipocyte CM (last bar) (Fig. 6Bi). CCl-2 (chemokine (c-c motif) ligand 2), also known as monocyte chemo-attractant protein-1 (MCP-1) and placental growth factor (Pgf) were approximately increased by 15-fold and 4-fold respectively by adipocyte CM (Fig. 6Bii & Biii). Metformin inhibited the expression of both the genes in ID8, when exposed to adipo CM or adipo Met CM (Fig. 6Bii & Biii). Adm (Adrenomedullin) was validated by an approximately 7-fold increase under adipo CM and was also inhibited by metformin treatments (Fig. 6Biv). Pfkl (Phosphofructokinase, liver, B-type) was up-regulated by 3-fold in ID8 cells exposed to adipo CM (Fig. 6Bv), while metformin treatment in basal media and adipo CM, induced Pfkl expression by 2-fold (Fig. 6Bv). Gadd45g (Growth arrest and DNA-damage-inducible 45 gamma) was seen to be up-regulated by adipo CM only by approximately 1.5 fold, while metformin treatments completely inhibited its expression (Fig. 6Bvi). The two down-regulated genes observed in the PCR-array were Serpin B2 (Serine [or cysteine] peptidase inhibitor, clade B, member 2) and FasL (Fas ligand, tumor necrosis factor superfamily, member 6). Interestingly, the results of Serpin B2 validation PCR were completely opposite to that of the PCR-array, as the Serpin B2 expression was seen to be induced by approximately 1.5 fold rather than inhibited (2-fold in the array) by adipo CM and was inhibited by various metformin treatments (Fig. 6Bvii). FasL was validated to be down-regulated in ID8 cells under adipo CM and was up-regulated by metformin (Fig. 6Bvii). Amongst the other up-regulated genes, Dsp (Desmoplakin), Pinx1 (PIN2/TERF1 interacting, telomerase inhibitor 1), nol3 (Nucleolar protein 3 [apoptosis repressor with CARD domain]), Snai1 (Snail homolog 1 [Drosophila]), Tinf2 (Terf1 [TRF^1^]-interacting nuclear factor 2), Ppp1R15a (protein phosphatase 1, regulatory (inhibitor) subunit 15A), Auraka (aurora kinase A) validated as being up-regulated by 2-fold or more by adipo CM in ID8 cells (Fig. S5A-D & I-K). Except for AurakA, which was increased, all others were inhibited by metformin treatments both to the differentiating adipocytes and ID8 under adipo CM. Sirt2 (Sirtuin 2 [silent mating type information regulation 2, homolog] 2 [S. cerevisiae]), IGFBP7 (Insulin-like growth factor binding protein 7), Birc3 (baculoviral IAP repeat-containing 3) and GSc (goosecoid homeobox) showed a trend towards higher expression (approximately 1.5 fold) with adipo CM (Fig. S5E, G, M, N). Metformin inhibited the expression of IGFBP7 and Birc3 (Fig. S5G & M), while it did not have any effect on the expression of GSc (Fig. S5N), and increased the expression of Sirt2 (Fig. S5E). No change was observed in the expressions of CcnD3 (Cyclin D3) and Ing1 (inhibitor of growth family, member 1) (Fig. S5F & L). Overall, adipo CM induced several genes in the ID8 ovarian cancer cells in order to promote the growth and survival, and metformin affected the expression of these tumor-promoting genes.

### MCP-1 and Pgf neutralization blocks adipocyte driven ID8 proliferation

**FIGURE 7:**
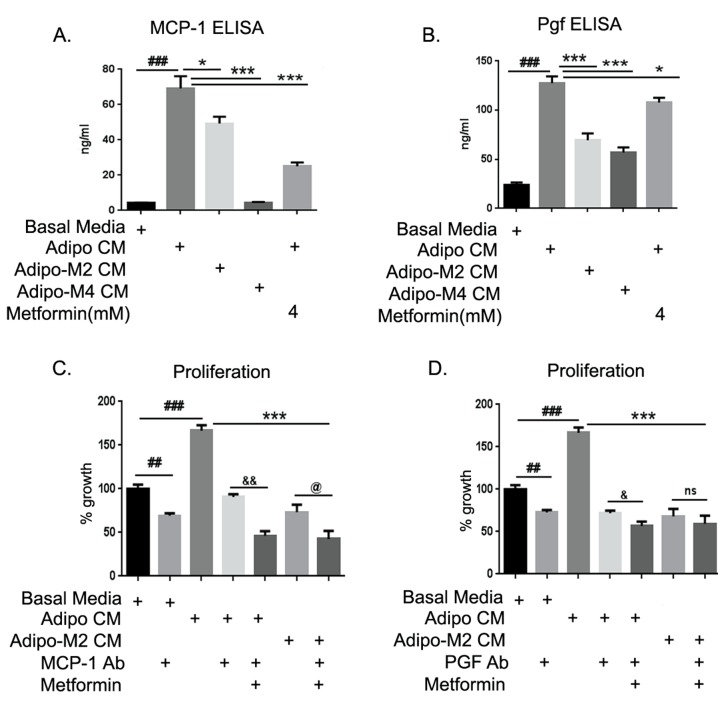
MCP-1 and Pgf neutralization blocked adipocyte potentiation of ID8 proliferation ELISA (enzyme-linked immunosorbent assay) for MCP-1 (monocyte chemo-attractant protein-1) and Pgf (placental growth factor) were performed in conditioned media (CM) from mature adipocytes and in supernatants from ID8 cells treated with various CM in absence or presence of metformin. Subtraction values from both ELISA readings were taken as quantitative concentration of cytokines being released by ID8 cells. (A) MCP-1 (###p < 0.001 compared to basal media; *p < 0.05, **p < 0.01, ***p < 0.001 compared to adipocyte CM [adipo CM]). (B) Pgf (###p < 0.001 compared to basal media; *p < 0.05, ***p < 0.001 compared to adipo CM). (C) ID8 cells plated in 96-well plate were exposed to the various CM in presence of the indicated treatments of MCP-1 antibody or Pgf antibody (D) with or without metformin, and increase in proliferation was estimated by MTT assay performed at 48 hours (##p < 0.01, ###p < 0.001 compared to basal media; ***p < 0.001 compared to adipo CM; &p < 0.05, &&p < 0.01 metformin and antibody combination compared to antibody alone in presence of adipo CM; @p < 0.05, ns: non-significant metformin and antibody combination compared to CM from adipocytes differentiated in presence of metformin 2mM [adipo M2 CM]).

MCP-1 and Pgf emerged as the most up-regulated growth factors in the ID8 cells in response to adipo CM exposure. The levels of both cytokines were estimated in the adipo CM (Fig. S6Ai & Bi) used to treat ID8 cells, in the supernatants of ID8 cells exposed to adipo CM and in presence of metformin and adipo Met CM (2 and 4mM) at 48 hours. The subtraction value of the two estimates was taken as the level being exclusively produced by the ID8 cells under the influence of adipo CM (Fig. 7A). MCP-1 expression was induced ~ 7-fold in ID8 cells by adipo CM exposure. Adipo Met CM (2 mM) induced significantly less expression of MCP-1, while adipo Met CM (4 mM) completely inhibited its production. Metformin treatment of ID8 cells was also able to attenuate the adipo CM induced MCP-1 production by ID8 cells (Fig. 7A). Adipo CM induced Pgf by ~ 5 fold in ID8 cells, while adipo Met CM showed significantly lower levels of Pgf in ID8 cells. Metformin treatment to ID8 was also able to inhibit the production of Pgf induced by adipo CM. Thus, soluble factors being produced by adipocytes are able to modulate ID8 cancer cells, which in turn produced increased amounts of these mitogenic, immunogenic and angiogenic factors. Metformin has the ability to act both on the differentiating adipocytes and their crosstalk with the cancer cells, in order to inhibit the expression of these pro-tumorigenic factors.

To assess the contribution of these chemokines on ID8 potentiated growth observed under the influence of adipo CM, respective neutralization antibodies were added to the various CM and ID8 cells before evaluating their effect on cell proliferation (Fig. 7C & D). The increased proliferation observed by adipo CM was inhibited by MCP-1 antibody. Combining the antibody with metformin further enhanced the inhibition of ID8 cell proliferation. Neutralization of MCP-1 antibody further inhibited proliferation of ID8 cells induced by adipo Met CM treatment (Fig. 7C). An exactly similar trend was observed in the presence of Pgf antibody (Fig. 7D). These data suggest that both MCP-1 and Pgf are crucial contributors to the adipocyte mediated growth potentiation of ID8 cells and that metformin treatment can inhibit their effect.

### Adipocytes influenced the cellular energetics of the ID8 cancer cell

**FIGURE 8:**
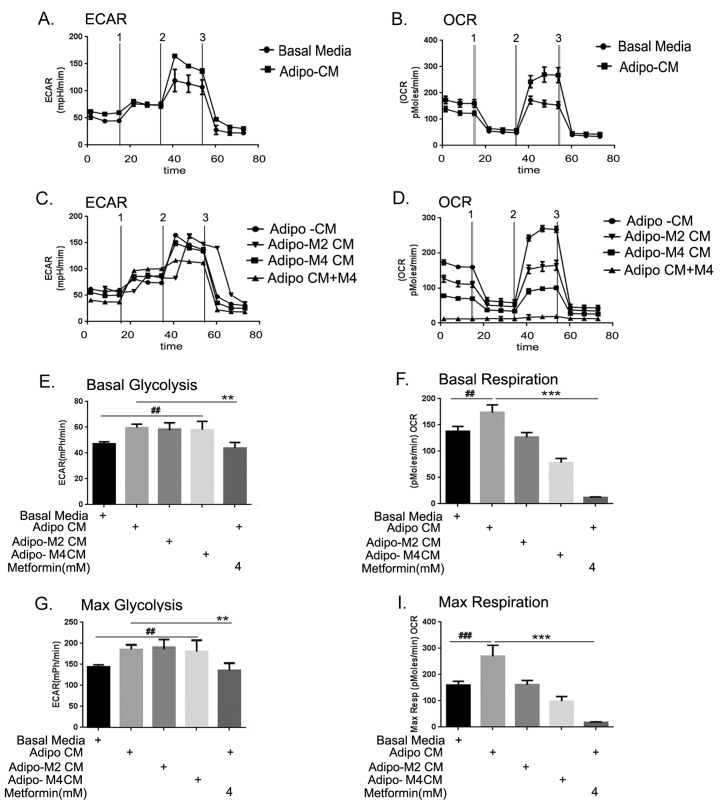
Adipocytes altered the cellular bioenergetics of the cancer cell (A) Extracellular acidification rate (ECAR) was measured as described in the Methods when challenged by (1) glucose, (2) oligomycin (an adenosine triphosphatase synthase blocker) and (3) 2-Deoxyglucose (an inhibitor of glycolysis). (B) Oxygen consumption rate (OCR) was measured as described in the Methods by a cell mitochondrial stress test when challenged by (1) oligomycin, (2) carbonylcyanide-p-trifluoromethoxyphenyl hydrazone (FCCP) (an electron transport chain uncoupler) and (3) rotenone/antimycin (an inhibitor of electron transport chain). (C) ECAR was measured in ID8 cells exposed to conditioned media from adipocytes differentiated in presence of metformin 2 or 4 Mm (adipo M2 CM or adipo M4 CM) and treated with metformin in presence of adipo CM. (D) OCR was measured in ID8 cells exposed to adipo M2 and M4 CM and treated with metformin in presence of adipo CM. (E) Basal glycolysis, is displayed as average of the first 3 resting state readings (##p < 0.01 compared to basal media; **p < 0.001 compared to adipo CM). (G) Maximum glycolytic capacity was calculated by normalizing at the oligomycin injections (##p < 0.01 compared to basal media; **p < 0.001 compared to adipo CM). (F) Basal respiration was taken as average of the first 3 resting state readings (##p < 0.01 compared to basal media; ***p < 0.001, compared to adipo CM). (H) Maximum respiration capacity was calculated by normalizing at the FCCP injections (###p < 0.001 compared to basal media; ***p < 0.001, compared to adipo CM).

Since proliferating cancer cells have been shown to metabolically adapt and shift towards using glycolysis as the main energy source [43, 44], we investigated if the exposure to adipo CM would result in any bioenergetic changes in the ID8 ovarian cancer cells. Using a Seahorse bioanalyzer, we found that ID8 cells exposed to the adipo CM showed higher glycolysis and oxidative phosphorylation (OX-PHOS) kinetics (Fig. 8A & B) compared to cells grown in basal media. Cells exposed to adipo Met CM did not show much change in the glycolysis; however, metformin treatment of ID8 cells showed slightly increased glycolysis (Fig. 8C). On the other hand, ID8 cells in the presence of adipo Met CM showed decreased OX-PHOS and direct treatment of metformin to ID8 cells completely inhibited OX-PHOS (Fig. 8D). The basal glycolytic values revealed that adipo CM induced glycolysis in ID8, but the increase was not statistically significant. Adipo Met CM also showed a similar pattern, while metformin treatment of the ID8 cells showed the same level of glycolysis as normal ID8 cells (Fig. 8E). On the other hand the basal respiration (OX-PHOS) was itself significantly increased in ID8 cells exposed to adipo CM compared to cells in basal media (Fig. 8E, 2nd bar vs 1st bar). Adipo Met CM or direct treatment to the ID8 cells significantly inhibited OX-PHOS (Fig. 8E, last 3 bars). The capacity for maximum glycolysis as well as respiration was significantly increased in the presence of adipo CM in the ID8 cells, though the increase in maximal respiration was much more significant compared to glycolysis (Figs. 8F & G). Thus, metformin treatment of the differentiating adipocytes and the ID8 cells did not affect glycolysis much in cancer cells but inhibited the capacity for increased OX-PHOS. These data indicate that the adipocyte environment is capable of inducing bioenergetic changes in the cancer cells even at short exposure times and metformin treatment modulates these changes.

## DISCUSSION

The role of the microenvironment in promoting the growth and metastasis of tumors is well recognized. The previously overlooked adipocytes are now considered vital components and regulators of the tumor microenvironment. They release various adipokines, cytokines, energy rich lipids and fatty acids which provide a fertile milieu for tumor cells to propagate [10, 45]. Ovarian cancer, a predominantly abdominal cancer, has been known to use omentum as one of its first sites of metastasis [2, 4]. A recent study demonstrated that the omental adipocytes attract the ovarian cancer cells to home to the omentum by secreting adipokines and subsequently providing a supply of fatty acids to meet the energy needs for rapid proliferation [6]. Any therapies that can disrupt the adipocyte-tumor cell interaction may be of significant benefit in hindering the initial step of ovarian cancer metastasis.

Our present study demonstrates that adipocyte derived CM promotes the growth, migration and invasion of the ID8 mouse ovarian cancer cells. These tumor promoting effects are associated with downstream function and gene expression and bioenergetic changes in the ovarian cancer cells. Studies have shown that breast adipocytes create a favorable microenvironment that promotes breast cancer growth [46, 47]. Furthermore, adipocytes have been shown to promote the growth or invasion of prostate [48], endometrial [49], lung [50], colon [9], gastric [51] and ovarian cancer cells [6]. Our findings of increased proliferation, migration and invasion of ovarian cancer cells under the influence of adipo CM are in line with these findings. These tumor promoting effects of the adipocytes can be significantly abrogated by metformin treatment through a number of mechanisms. Our study supports both the direct and indirect effects of metformin, showcasing the indirect effects whereby metformin is retarding the growth of ovarian cancer cells that is being specifically promoted by the adipocyte component of the microenvironment. Metformin can act at multiple levels regulating factors that arise systematically as well as those produced by the cancer cell itself to reduce and prevent cancer growth [30, 52].

Metformin completely inhibited the differentiation of preadipocytes to adipocytes by attenuating the key transcription factors. CEBP and PPAR are considered early adiopgenesis genes. They are thought to be involved in growth arrest that is a prerequisite for differentiation, and they also trans-activate other adipocyte specific genes [42]. SREBP1 participates in cholesterol metabolism and activation of other adipocyte gene expression [42]. ATP-citrate lyase is a terminal phase enzyme that is involved in de novo lipogenesis [42]. SREBP1, CEBPα and CEBPß were inhibited by metformin treatment without altering the levels of ATP citrate lyase or PPARγ expression (Figs. 3 & 4). The specific inhibition of some, but not all, adipogenic transcription factors suggests that the regulation of adipogenesis in the adipocytes by metformin is specific. Metformin also induced AMPK activation in those cells as observed by higher levels of phosphorylation of AMPK and its downstream target ACC. We have previously shown that activation of AMPK by AICAR (5-Aminoimidazole-4-carboxamide ribonucleotide) results in inhibition of adipogenesis in 3T3L1 preadipocytes along with inhibition of adipogenic transcription factors [53]. Thus, activation of AMPK may play a regulatory role in lipid metabolism.

It was recently reported that adipocytes can promote lipid accumulation in ovarian cancer cells by providing fatty acids [6]. The underlying mechanism was suggested to be an up-regulation of fatty acid transporter gene, fatty acid binding protein 4 (FABP4), in the ovarian cancer cells positioned next to adipocytes to facilitate the transport of fatty acids [6]. In our experimental model, the adipo CM induced lipid droplet formation in the ID8 cells. This lipid accumulation was inhibited to various degrees when either the adipocytes or the cancer cells were treated with metformin (Fig. 5). Metformin has been shown to decrease intracellular lipid levels in various cells such as hepatocytes, preadipocytes, skeletal muscle and macrophages [41, 53-55]. Song et al. [55] also demonstrated that metformin can reduce fatty acid induced lipid accumulation in a macrophage cell line by downregulation of FABP4 and up-regulating CPT-1 (Carnitine palmitoyltransferase 1). FABP4 has been shown to play a critical role in uptake of fatty acids and accumulation of lipid [56], while CPT-1 is a rate limiting enzyme in the fatty-oxidation process [57]. Thus it can be hypothesized that along with inhibition of de novo lipogenesis, metformin may regulate the transport of fatty acids supplied by the adipocytes into the tumor cells. Another mechanism by which metformin and AMPK activation may impede lipid accumulation in cancer cells involves the inhibition of lipolysis, which is responsible for the release of fatty acids from the adipocytes. Bourron et al. [58] have found that metformin inhibits stimulated lipolysis in human adipocytes and causes inactivation of hormone sensitive lipase translocation to the lipid droplet. Activation of hormone sensitive lipase has been associated with increased levels of free fatty acids in adipocytes and their uptake by cancer cells [6].

The mature adipocytes are now recognized as active endocrine organs that not only function as energy depots for the body but also secrete factors that are known to regulate immune response, vascular diseases and cancer [10]. Some of these secretary factors include adipokines, cytokines and chemokines which enhance tumor cell proliferation, migration and invasion [6, 47]. We observed that these tumor enhancing abilities of the adipocytes' secretory products were accompanied by gene expression changes in the ID8 cells (Fig. 6). Twenty-two genes were differentially modulated by a minimum of 2-fold and spanned multiple pathways including angiogenesis (Ccl-2, Pgf), apoptosis (FasL, Birc3, Nol3), cell cycle (MKi-67, AuraKa, CcnD3, Stmn1), DNA damage and repair (Gadd45g, Ppp1r15a), cell senescence (SerpinB2, IGFBP7, Ing1), epithelial-mesenchymal transition (Dsp, Snai1, GSc), hypoxia (Adm), metabolism (Pfkl) and telomere and telomerase (Pinx1, Sirt2, Tinf2) pathways.

The top 6 genes up-regulated by 5-fold in ID8 cells when exposed to adipo CM included Ki-67, MCP-1, Pgf, Adm, Pfkl and Gadd45g; on the other hand, 2 genes (SerinB2 and FasL) were found to be down-regulated. Ki-67 is a cellular proliferation marker shown to be highly expressed in almost all proliferating tumor cells and its expression (Ki-index) is often co-related with cancer progression [59]. We have previously shown metformin treatment to inhibit Ki-67 in ovarian tumors *in vivo* [26]. MCP-1, one of the most up-regulated genes in our model, has been shown to promote proliferation, migration and invasion of cancer cells. It has also been demonstrated to attract tumor-associated macrophages and other inflammatory cells towards the tumors [60, 61]. MCP-1 has also been implicated in adipogenesis and has been shown to be produced by adipocytes [62]. Nieman et al. [6] also has identified MCP-1 as one of the highly expressed cytokines in the omental adipo CM. Pgf belongs to the VEGF family and signals through VEGFr1 (Flt-1) [63] to promote angiogenesis [47, 64]. It has been shown to be highly expressed in cancers like renal, colon, pancreatic, melanoma, colorectal, lung, gastric and breast [65-70]. One study in ovarian cancer has shown increased Pgf plasma levels to be associated with poor prognosis [71]. On the other hand, a variant Pgf (Plgf2) has been shown to inhibit tumor growth by blocking VEGFa [72]. In our current study, we found that adipocytes secreted high MCP-1, but did not appear to produce Pgf. Both MCP-1 and Pgf were induced in the ID8 cancer cells by adipo CM (Fig. 7).Both MCP-1 and Pgf expression were inhibited by metformin. Complete inhibition of ID8 growth was observed after neutralization of these 2 proteins by their respective antibodies.

Adm, another gene upregulated by adipocytes in the ID8 cells, encodes a peptide that belongs to the calcitonin superfamily and is induced by various cytokines and hypoxic conditions. It has been shown to induce proliferation of breast and endometrial cells and promote vasculogenesis [73, 74]. PfKl is a key enzyme in the glycolysis pathway and has been associated with increased glycolytic capacity of the cell [75]. Gadd45g is a gene that is expressed under stress conditions or DNA damaging conditions and activates the p38/c-Jun N-terminal kinases pathway [76]. It is a putative tumor suppressor gene and is down-regulated in multiple cancers [77]. Gadd45g was surprisingly up-regulated in ID8 cells by adipo CM and completely inhibited by metformin.

FasL, a membrane protein belonging to the tumor necrosis factor family [78], was down-regulated by adipo CM and was up-regulated by metformin. FasL induces apoptosis, but also plays a vital role in deterring autoimmune response in immune privileged sites. Due to these different actions, it is believed to have a dual role in cancer progression [79, 80]. In ovarian cancer, high FasL expression and secretion have been linked to prevention of immune cell mediated cell death [80, 81]. In our model, it is possible that the inhibition of FasL by adipocytes indicates a means of subduing the apoptosis pathway while metformin helps to reinstate it. We have not measured the secretory FasL, which plays a greater role in modulating the immune escape of tumor cells and our *in vitro* system is devoid of immune cell presence. Serbinb2, also known as PAI-2 (Plasminogen activator inhibitor-2), was the second down-regulated gene in the array, but on validation we found it to be 1.5 fold up-regulated in ID8 cells exposed to the CM. Metformin treatments, both to the differentiating adipocytes or to the ID8 cells, resulted in its decreased expression. Serbinb2 is an inhibitor of uPA (urokinase plasminogen activator) and has been shown to be expressed in adipocytes among other cells and promote adipogenesis [82]. It also plays a role in inflammation and cisplatin chemo-resistance [83]. Three genes (CCND3, STMN1 and Ing1) were not increased on validation; this discrepancy could be explained by the difference in the primers used for validation or a false positive result in the array.

One adaptation by the cancer cells to meet the demands of increased proliferation and to overcome adverse conditions is to utilize glycolysis as the primary source of ATP generation in lieu of mitochondrial oxidative respiration (OX-PHOS). It was interesting to observe that the adipo CM enhanced both glycolysis and OX-PHOS in ID8 cells. While cancer cells are believed to be highly glycolytic as per the ‘Warburg effect’ [84], the implications of an increased OX-PHOS state has not been previously elucidated in cancer cells. Increased glycolysis supports the demand of cancer cells for increased macromolecules [43]. The increased OX-PHOS can be postulated to be occurring due to the excess of fatty acids being supplied by adipocytes, which is oxidized in mitochondria through the OX-PHOS pathway. Thus the cues provided by the adipo CM provides dual advantage to the cancer cells in terms of achieving higher glycolysis as well as OX-PHOS capability, which could be aiding faster proliferation and higher energy availability to the cell. Metformin did not further increase glycolysis above that observed with adipo CM but did significantly inhibit OX-PHOS. This is in agreement with other studies where metformin has been shown to enhance glycolysis but inhibit OX-PHOS [85-87]. Whether decreased OX-PHOS contributes to the inhibition of ID8 proliferation by metformin remains to be determined. Interestingly, rapamycin, an inhibitor of mTOR pathway, showed similar effects as metformin in obese conditions and aging, however, rapamycin treatment ameliorates lactate production, which would indicate inhibition of glycolysis [88, 89], while metformin has been reported to induce glycolysis. Overall, these studies where by adipocytes contribute nutritionally to the microenvironment to influence the bioenergetics of tumor cell are supported by the paradigm of obesity (excess adiposity) being the provider of surplus nutrition which results in aggressive tumor growth and upregulation or inhibition of nutrition sensitive signaling pathways like mTOR and AMPK [28, 29]. Metformin has the ability to not only regulate cellular energetics by regulating mitochondrial activity but also by the signaling that controls the energy state of the cell.

Our present work is completely based on an *in vitro* approach, which is one of the limitations of our study. These *in vitro* techniques are well used in research and have recently been validated by *in vivo* models. A recent comprehensive report by Clark et al. [90] establishes the importance of immune cell rich milky spots in conjunction with adipose tissue (omentum and spleen) for metastasizing of ovarian cancer. Overall, our data strongly supports the role of adipocytes in promoting ovarian tumor growth, particularly by the production of various adipokines and their consequent effects on cancer cells that encompass functional, genotypic and bioenergetic changes, which collectively enhance the growth and spread of ovarian tumors. Metformin, by regulating adipogenesis and altering adipokines and their stimulant effects on ovarian cancer cells, is a promising candidate drug to disrupt the adipocyte-cancer cell crosstalk and mitigate tumor progression.

## MATERIALS AND METHODS

### Reagents and antibodies

MTT and anti-Pgf-1 were purchased from Sigma (St. Louis, MO, USA). Phospho-AMPKα, phospho-ACC, total-AMPKα, total-ACC, PPARγ and ATC antibodies were from Cell Signaling (Danvers, MA, USA). Anti-CEBPα and Anti–CEBPß were purchased from Santa Cruz Biotechnology (Santa Cruz, CA, USA). Anti-SREBP1 was from BD Pharmingen (San Jose, California, USA) and anti-MCP-1 was from Novus Biologicals (Littleton, CO).

### Tissue culture

3T3L1 mouse preadipocytes were obtained from Dr. Giri's laboratory. The cells were cultured in DMEM containing 10% (v/v) fetal calf serum (FCS) and used till passage 8. ID8 mouse ovarian cancer cells were a gift from Dr. Keith Knutson, (Vaccine & Gene Therapy Institute of Florida, Port Saint Lucie, FL) and were maintained in RPMI media containing 10% (v/v) fetal bovine serum (FBS). All media and FCS were purchased from HyClone-ThermoScientific (Waltham, MA, USA) or Gibco Life Technologies (Grand Island, NY, USA). FBS was purchased from BioAbChem (Ladson, SC, USA).

### Adipocyte differentiation

Differentiation was initiated using chemicals from Cayman Chemicals (Ann Arbor, MI, USA) as described before [53]. Briefly, 3T3L1 preadipocytes were cultured in DMEM containing 10% (v/v) FCS and plated in 100 mm dishes. Once the cells reached confluence (48-72 h later), cells were induced to differentiate by replacing media with induction media containing 10% (v/v) FBS, insulin (1.7 μmol/L), 3-Isobutyl-1-methylxanthine (0.5 mmol/L), and dexamethasone (DEX, 1 μmol/L). After 3 days, induction media was replaced with media containing insulin. Media was changed every alternate day. Differentiation was estimated by performing Oil Red O staining. Adipocyte differentiation was observed at 70-80% by days 7-9. For experiments with metformin treatments, metformin was added to the respective media either from day 0 or day 3 of differentiation. At differentiation (day 8), cells were washed with sterile phosphate buffered saline (PBS) twice and cultured for an additional 48 hours in regular DMEM media. CM was collected filtered and stored at −20^°^C till further use.

### Oil red O staining

Predifferentiated and differentiated adipocytes were washed twice with PBS and fixed in formaldehyde (3.7%) for 1 hour. Oil red O solution (0.2% (w/v) in 60% (v/v) isopropanol) was applied for 30 minutes. Cells were then washed with water and slides were dried by placing them under the flow hood and examined under bright-field. In order to quantitate the adipose conversion, 0.2 ml of isopropanol was added to the stained cells and absorbance was measured using a spectrophotometer at 510 nm [53].

### Metformin treatments

The effect of metformin was examined using two approaches. The first one involved directly adding metformin in the media during adipocyte differentiation. On day 7, adipocytes were washed with sterile PBS and replaced with fresh complete media without metformin. Forty-eight hours later, CM was collected, filtered and stored at −20°C till further use. CM was used to examine the effect of adipo CM and adipo Met CM on ID8 cell proliferation, migration and invasion. The second approach involved direct treatment of ID8 cells with metformin in presence of adipo CM. In the first approach, we were assessing the production of soluble factors and lipids by the maturing adipocytes under metformin treatment, while in the second approach we were studying the ability of metformin to block the stimulatory effects of adipocyte soluble factors on the cancer cells.

### Proliferation assays

To determine the effect of adipocytes on proliferation of ID8 cells, 4000 cells per well were plated in 96-well plates in RPMI media containing 10% (v/v) FBS overnight. Media was replaced the next day with a mixture of complete RPMI media and the CM in various ratios (75:25; 50:50; 0:100). For all subsequent experiments an optimal ratio (50:50; RPMI:CM) was used. Metformin treatment to the ID8 cells was done at the same time of media replacements. MTT assay was performed at 24, 48 and 72 hours post-treatment as described earlier [19].

### Scratch migration assay

ID8 cells were grown to confluence in 24-well plates and kept in low serum conditions (0.2%) overnight. A scratch was created in the middle of the well with a sterile 200μl tip, and the media was replaced with the respective treatments. Micrographs were collected at 0 and 24 hours. The pixel distance between the gaps was measured at a predetermined point at each time point. The distance covered was subtracted from the start time point to estimate the distance covered by the migrating cells [91].

### Boyden chamber migration and invasion assay

Cell migration and invasion were measured as previously described [92] with some modifications. Briefly, ID8 cell suspension (500 μl, 2.5×10^4^ cells) was seeded on the top of Matrigel-coated (invasion assay) 8-μm pore diameter transwell plate from BD Biosciences (San Jose, California, USA). The CM from differentiated adipocytes in presence or absence of metformin was placed in the lower chamber. For exogenous metformin treatment of ID8 cells, the ID8 cells were pretreated with metformin overnight before seeding in the matrigel chamber. For these studies we exposed the ID8 cells to 100% CM (0:100; RPMI:adipo CM) to promote significant invasion. Cells invading the lower chamber were stained with 0.5% crystal violet (60% PBS, 40% ethyl alcohol) and counted with an inverted microscope. The invaded cells were also lysed in isopropanol to estimate the invasion by colorimetric assessment by reading the optical density at 575 nm. The results from at least two independent experiments in triplicate are presented.

### Western blots

Differentiating adipocytes in the presence or absence of metformin at indicated time points were washed with PBS and lysed in 500 μl of protein lysis buffer (50 mM Tris-HCl, pH 7.5, 250 mM NaCl, 5 mM ethylenediaminetetraacetic acid (EDTA), 50 mM NaF, and 0.5% Nonidet P-40) containing a protease inhibitor cocktail (Sigma, St Louis, MO, USA). ID8 cells under CM exposure were also similarly harvested at 24 hours. Supernatant was separated and protein concentration was estimated by the Bradford method (BioRad, Hercules, CA, USA). Immunoblot analysis was performed using various antibodies as previously described [19].

### Bodipy stain

ID8 cells were grown in RPMI:adipo CM (50:50) for 72-96 hours in 8-well chamber slides [93]. Cells were washed, fixed in paraformaldehyde and stained with Bodipy 493/503 (Life Technologies, Grand Island, NY, USA) at 1:1000 dilutions for 15 minutes as per the manufacturer's instructions. Cells were counterstained with Hoechst 33342 and mounted in vectashield. The lipid droplets stained green were visualized with a fluorescent microscope at 40x magnification.

### Cancer pathway finder PCR array

The Mouse Cancer PathwayFinder™ RT² Profiler™ PCR array (SA Biosciences, Valencia, CA, USA) was performed to obtain the expression profiles of 84 genes pertaining to 9 cancer pathways. ID8 cells were treated under various experimental conditions for 24 hours and processed for RNA isolation. An equal amount of RNA (2 μg) was used to synthesize complementary DNA (cDNA) using RT^2^ First Strand Kit (Qiagen, Valencia, CA, USA) and cDNA was applied to the PCR Array plate along with RT master mix. PCR was run in BIO RAD CFX96TM real time PCR instrument (Hercules, CA, USA) and data was analyzed using web based RT2 Profiler PCR Array Data Analysis software (http://pcrdataanalysis.sabiosciences.com/pcr/arrayanalysis.php, SA Biosciences). Five housekeeping genes (glucuronidase β, HPRT1 [hypoxanthine phosphoribosyltransferase 1], HSP90 alpha [heat shock protein], GAPDH [glyceraldehyde 3-phosphate dehydrogenase] and β-actin) were included in the array to minimize functional biases.

### Quantitative PCR

Real-time PCR was performed with cDNA generated from RNA isolated from ID8 cells under various CM exposures and metformin treatment for 24 hours as described previously [94]. All primers used were purchased from RealtimePrimers.com (Ekins Park, PA, USA). PCRs were performed using BIO RAD CFX96TM real time PCR instrument (Hercules, CA, USA).

### Cellular bioenergetics

Measurements of oxygen consumption rates (OCR) and extracellular acidification rate (ECAR) were performed using the Seahorse XFe Extracellular Flux Analyzer (North Billerica, MA, USA). Briefly, 5 × 10^4^ of ID8 cells were plated in XF96 96-well microplates coated with Cell-Tak from BD Biosciences (San Jose, California, USA). After a 3-hour incubation, media was replaced with the various CM and metformin as indicated in the volume of 100 μL per well. Cells were incubated for 24 hours at 37°C at 5% CO_2_. The following day the media was removed and cells were washed twice with either XF medium with 3% glucose containing cell mitochondrial stress test media or glucose stress test media without glucose before adding 175 μL of cell mitochondrial or glucose stress test media in each well. The plate was incubated for an additional 1 hour at 37°C without CO_2_ to allow temperature and pH to reach optimal assay conditions. A 3-minute mix and 3-minute reading cycle was used to obtain the OCR and ECAR readings. During this time, various compounds were injected which altered the metabolic response of the cells. The compounds for the OCR measurements included oligomycin at a final concentration of 1μM as the first injection. The second injection was the electron transport chain stimulator, carbonilcyanide p-triflouromethoxyphenylhydrazone (FCCP) at a final concentration of 250 nM. The final injection was a combination of 2 inhibitors, Antimycin A and Rotenone, both at a final concentration of 1 μM. The first compound used for the ECAR measurement was glucose at a final concentration of 10 mM followed by oligomycin at a final concentration of 2 μM. The last injection was 2-deoxyglucose (2DG), a glucose analog which interrupts glycolysis, at a final concentration of 100 mM [95]. Data obtained was normalized with cell number counts.

### Statistical analysis

Data were statistically analyzed using two-tailed *t*-tests or unpaired *t*-tests (GraphPad Software, La Jolla, CA, USA).

## SUPPLEMENTARY FIGURES


