# Supplementary material for: Metformin limits the adipocyte tumor-promoting effect on ovarian cancer
Source: Oncotarget. 2014 May 26;5(13):4746–64. doi: 10.18632/oncotarget.2012 (PMC4148096; doi:10.18632/oncotarget.2012)
Supplement: Supplementary file 1 [file oncotarget-05-4746-s001.pdf]

Metformin limits the adipocyte tumor-promoting effect on ovarian cancer – Tebbe et al

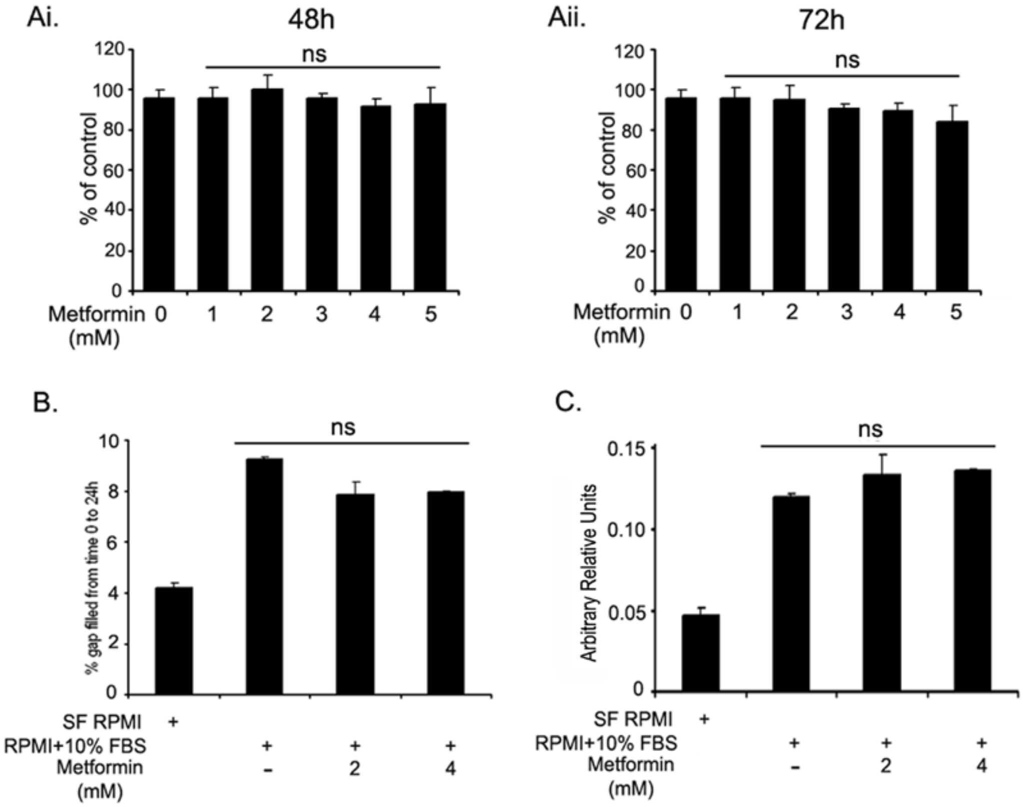

FIGURE S1: Low doses of metformin do not have a direct effect on ID8 cells. Four thousand ID8 cells per well were plated in 96-well plates and treated with indicated concentrations of metformin (1-5 mM) and cell numbers were assessed by MTT assay at 48 **(Ai)** and 72 hours **(Aii)** (ns: non-significant compared to untreated cells). **(B)** Migration potential of ID8 cells was assessed in presence of serum after treatment with metformin (2-4 mM) by scratch assay. **(C)** Invasion capability of ID8 was induced by serum with and without metformin using the Boyden Chamber (ns: non-significant compared to untreated cells).

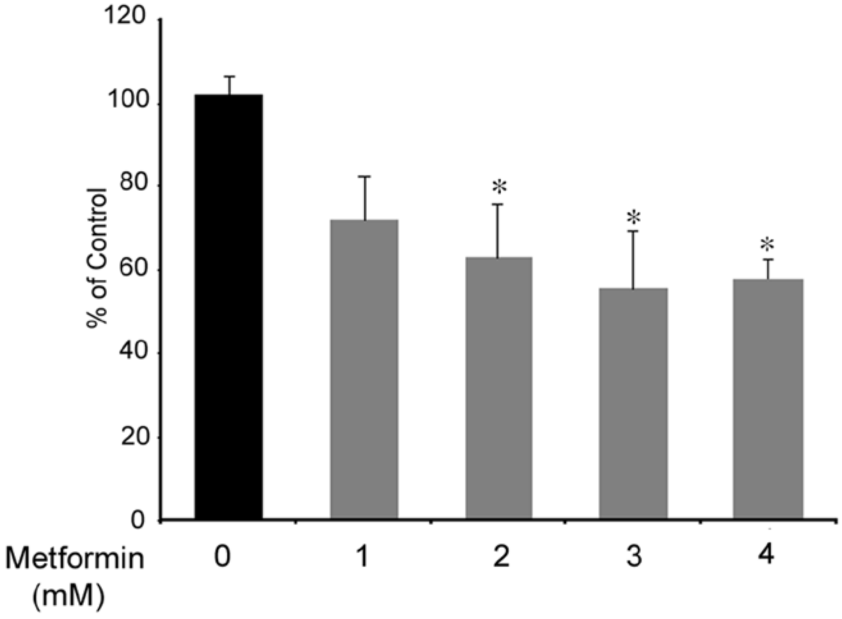

FIGURE S2: Metformin inhibited growth of preadipocytes. 3T3L1 preadipocytes were treated with various concentrations of metformin (1-5 mM) and proliferation was assessed by MTT assay after 72 hours (*p <0.005 compared to untreated).

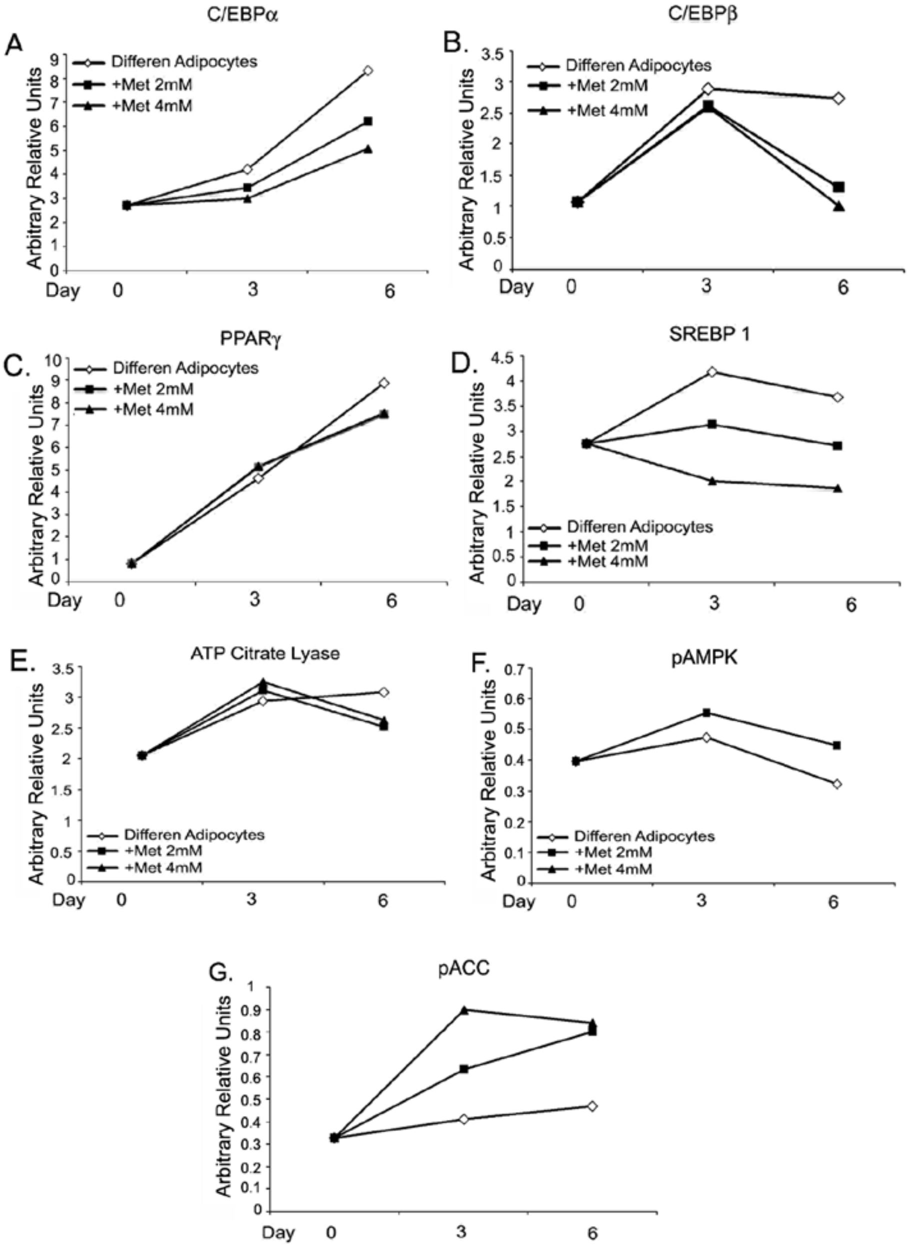

FIGURE S3: Metformin inhibited adipogenesis regulating transcription factors. Western blot images were quantified by reading the band densities using the Quantity One 1-D densitometric analysis tool from BioRad (Hercules, CA). The density of protein band was divided by its respective beta-actin or total protein band density for normalization and is represented as arbitrary units. **(A)** CCAAT-enhancer binding protein alpha (CEBPα), **(B)** CCAAT-enhancer binding protein beta (CEBPß) **(C)** Peroxisome proliferator-activated receptor gamma (PPARγ) **(D)** Sterol regulatory element-binding protein -1 (SREBP1), **(E)** adenosine triphosphate (ATP) Citrate Lyase, **(F)** pAMPK (phosphorylated adenosine monophosphate activated kinase**) and (G)** pACC (phosphorylated acetyl CoA carboxylase).

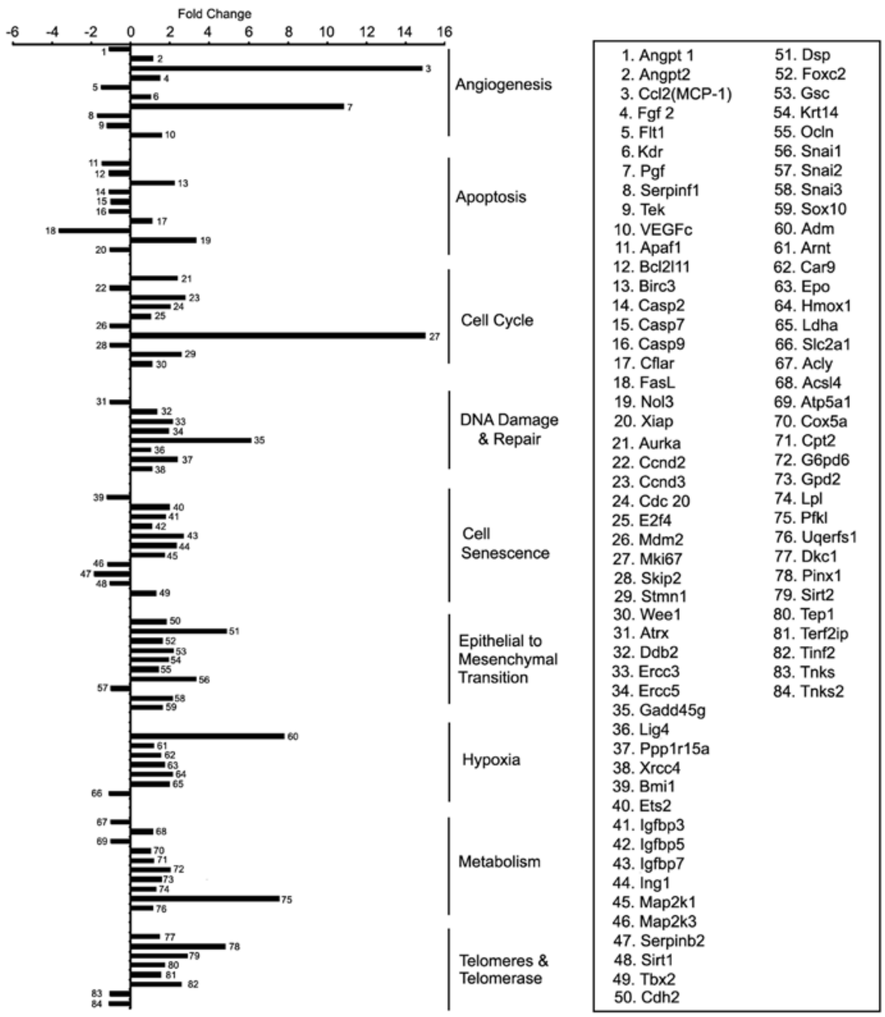

FIGURE S4: Adipocyte conditioned media modulated the expression of tumor associated genes in ID8 cells. A. Bar graph representation of the fold change (adipocyte conditioned media exposed ID8 cells/ID-cells) observed in the 84-cancer related genes investigated in the Mouse Cancer PathwayFinder™ RT² Profiler™ PCR Array (SA Biosciences, Valencia, CA). The genes have been arranged by the various pathways they represent.

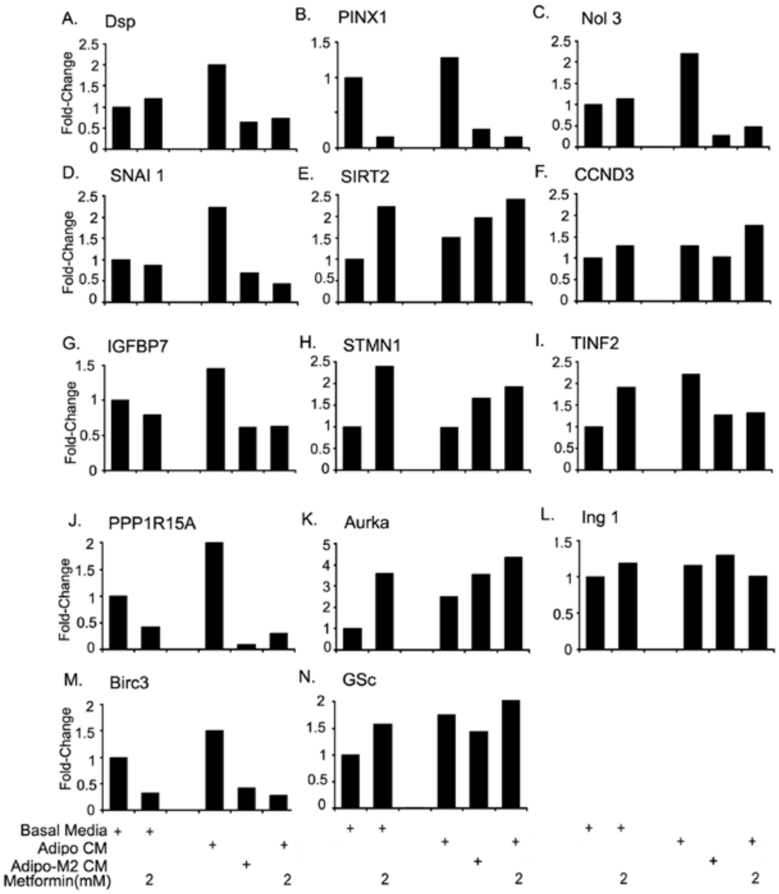

FIGURE S5: Metformin modulated the expression adipocyte induced tumor promoting genes. ID8 cells were exposed to basal media with or without metformin and in adipocyte conditioned media (adipo CM) with or without metformin for 24h before extracting RNA and synthesizing complementary DNA to validate the 22 genes that were modulated 2-fold or more under adipo CM. The 6 top up-regulated and 2 down-regulated genes were presented in Figure 6. The rest of the 14 genes are depicted here. **(A)** Dsp (Desmoplakin); **(B)** PINX1 (PIN2/TERF1 interacting, telomerase inhibitor 1); **(C)** nol3 (nucleolar protein 3 [apoptosis repressor with CARD domain]); **(D)** Snai1 (Snail homolog 1 [Drosophila]); **(E)** SIRT2 (Sirtuin 2); **(F)** CCND3 (CyclinD3); **(G)** IGFBP7 (insulin growth factor binding protein 7); **(H)** STMN1: **(**Stathmin 1/oncoprotein 18); **(I)** TINF2 (Terf1 [TRF1]-interacting nuclear factor); **(J)** Ppp1R15A (protein phosphatase 1, regulatory (inhibitor) subunit 15A); **(K)** AurakA (aurora kinaseA); **(L)** Ing1 (inhibitor of growth protein 1); **(M)** Birc3 (baculoviral IAP repeat-containing 3); **(N)** GSc (goosecoid homeobox).

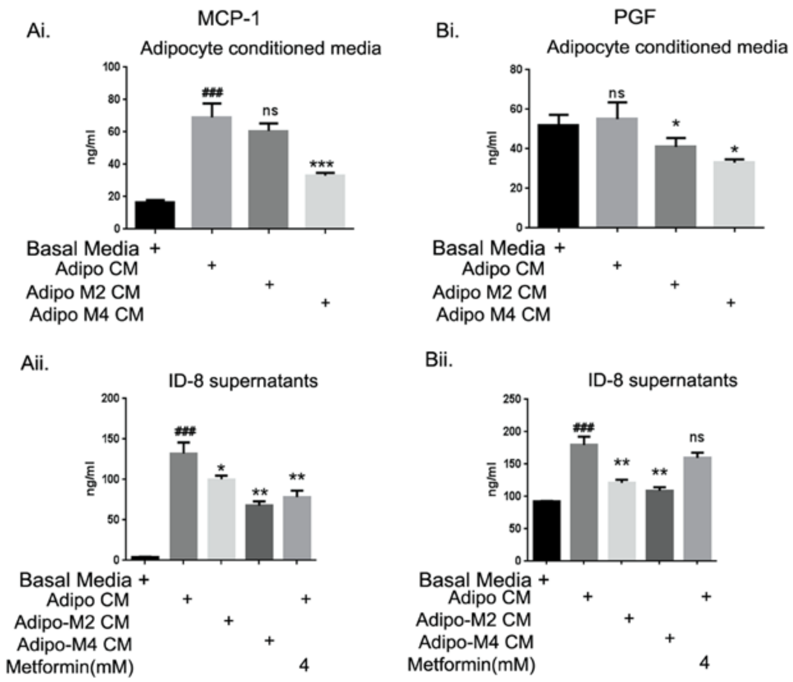

FIGURE S6: Metformin inhibited adipocyte induced expression of MCP-1 and Pgf: **(Ai & Bi)** Adipocyte conditioned media (adipo CM) and adipo metformin CM were subjected to ELISA (enzyme-linked immunosorbent assay) for MCP-1(monocyte chemo-attractant protein-1) **(Ai)** and Pgf (placental growth factor) **(Bi)** **(**###p < 0.001, compared to preadipocytes; ***p < 0.001 compared to adipo CM; *p < 0.05 compared to adipo CM; ns= non-significant). Supernatant collected from ID8 cells after being exposed to various CM with or without metformin were subjected to ELISA for MCP-1**(Aii)** and Pgf **(Bii)** (###p < 0.001, basal media; **p < 0.01, *p < 0.05, compared to adipo CM; ns=non-significant compared to adipo CM).
